# Eating Disorders and Nutritional Beliefs, Trends or Practices

**DOI:** 10.3390/nu15092058

**Published:** 2023-04-25

**Authors:** Ata Ghaderi

**Affiliations:** Division of Psychology, Department of Clinical Neuroscience, Karolinska Institutet, 171 77 Stockholm, Sweden; ata.ghaderi@ki.se; Tel.: +46-8-524-832-48

This Special Issue of *Nutrients* on “Eating disorders and nutritional beliefs, trends or practices” contains ten empirical papers that cover various aspects of the topic.

Brytek-Matera and colleagues [1] investigated the relationship between disordered eating attitudes, self-esteem, physical activity, and orthorexia nervosa in two samples of young adults from Poland and Italy. Given the current non-diagnostic status of orthorexia nervosa, most studies on orthorexia nervosa are correlational in terms of methodology, and replications in different samples as well as with various measurements methods are necessary to help us understand the nature of this condition. Orthorexia nervosa is characterized in different ways: while overconcern about healthy eating is one of its core characteristics, increased frequency of physical exercise and its overevaluation are still matters of debate. The study [1] found several associations in relation to specific aspects of the measure of orthorexia nervosa that corroborate previous findings, but, more importantly, they found that young adults with higher scores on a measure of orthorexia nervosa also reported more vigorous-intensity physical activity than those with lower indications of orthorexia nervosa.

The second paper, by Halbeisen et al. [2], is a case-matched, retrospective comparison of gender differences in eating disorder (ED) treatment outcomes. Men remain under-represented in research on EDs, and we need to know how men respond to treatments for EDs compared to women. Thus, they investigated the treatment outcomes for men and women (*n* = 400) by matching them based on age, ED diagnosis, and length of treatment. Men with anorexia nervosa and binge eating disorders had more favorable outcomes than women (i.e., better weight gain as well as improved general and ED-specific psychopathology in anorexia nervosa, and more weight loss in men with binge eating disorders). The outcomes for men with bulimia nervosa or “other EDs not otherwise specified” were virtually the same as for women with corresponding diagnoses. This study adds more specific knowledge to the literature on the outcomes of ED treatments for men compared to women. The outcomes for men do not seem to be more favorable to women independent of the diagnosis.

The third paper, by Steinmann and colleagues [3], is another line of work on orthorexia nervosa. As they argue, nutritional beliefs play an important role when it comes to food choice. These authors investigated what kinds of food are considered to be comforting in the context of stress. More specifically, they examined whether the participants (*n* = 175) believed that only healthy foods relieve stress, and if such beliefs were related to orthorexia nervosa. Through principal component analysis and latent profile analysis, they identified eight distinct groups, of which one (8% of the sample) held such beliefs. This group was also characterized by higher scores on a measure of orthorexia nervosa. They conclude that nutritional beliefs in orthorexia nervosa are not only about the somatic consequences of some foods, but also their psychological consequences. Future research should investigate the relationship between these beliefs and orthorexic behaviors, as well as their role in the diagnosis and treatment of orthorexia nervosa.

The fourth paper, by Gwioździk et al. [4], sheds light on another aspect of nutritional beliefs as well as trends and their relationship with EDs. Dietary consultations and recommendations are becoming increasingly common; some diets introduce various forms of restraint that might increase the risk for EDs. These authors investigated the occurrence of symptoms of EDs or orthorexia nervosa among young women who follow a traditional, vegetarian, or therapeutic (low FODMAP) diet. Among 420 females (19 to 30 years old) following different diets, they found that uncontrolled and emotional eating were the least common among those on a vegetarian diet, while symptoms of orthorexia nervosa were the most common among those on a low FODMAP diet. They conclude that screening for EDs should be considered as a first step before any dietary recommendations are provided.

The fifth paper is the initial work of developing and investigating the psychometric properties of the Eating-Related Eco-Concern (EREC) questionnaire by Qi and colleagues [5], given the distress experienced due to climate change and its association with mental health but lack of a relevant measure of such concerns in relation to disordered eating. The 10-item EREC loaded on a single factor and showed expected associations with eco-concern as well as a measure of disordered eating in a sample of 224 participants. The internal consistency of the ERED was high, and non-significant sex differences emerged.

The sixth paper, by Ghaderi and Welch [6], investigated the frequency of use of appearance- and performance-enhancing drugs and supplements (APEDSs) in an anonymous online sample of 824 young men (15–30 years old) along with their self-reported ED symptoms. The use of APEDSs has become a worrisome nutritional trend among young people, and very little is known about their use in relation to ED symptoms. A marked proportion of the participants (16.1%) reported the regular use of APEDs (at least once a week). Dietary restraint, binge eating, compensatory vomiting, and excessive exercise, as well as a drive for muscularity, predicted the use of APEDSs. In addition, a larger proportion of participants who identified as heterosexual reported using APEDSs (34%) compared to those who identified themselves as homosexual (25%) or other (23.7%). Taken together, the results suggested that the use of APEDSs might be more related to a drive for muscularity and sexual orientation than to symptoms of EDs.

The seventh paper, by Gabloffsky et al. [7], used an animal model to experimentally investigate the relationship between food restriction, food-anticipatory activity, and changes in activity due to circadian rhythm to further our understanding of hyperactivity in anorexia nervosa. They induced starvation, by restricting access to food in adolescent mice, until a 20% reduction in weight was reached and maintained for two weeks. The animals displayed an increase in locomotor activity for a few hours before food presentation (i.e., food-anticipatory activity). Hyperactivity was only observed among chronically starved animals, although amenorrhea was present in virtually all of the cohorts. The chronically starved mice displayed a decrease in circadian-rhythm-related activity at nighttime. Gabloffsky and colleagues [7] conclude that chronic starvation in mice mimics behaviors observed in anorexia nervosa, and that food-anticipatory activity might be a direct consequence of starvation. They suggest that the changes related to circadian activity might be an important mechanism in the pathophysiology of anorexia nervosa, which justifies further studies using this and similar models.

The eighth study, by McLeand et al. [8], investigated the psychometric properties of the 26-item version of the Eating Attitude Test (EAT) in a sample of vegetarians (*n* = 278), vegans (*n* = 580), and omnivores (*n* = 413). Confirmatory analyses of the current relevant models showed an inadequate fit between data and models, as well as poor psychometric properties. In addition, the poor test–retest reliability of both the full and shortened version puts its use among vegetarians and vegans into question. This is an important study, as measurements are the bases of our knowledge, and the use of assessment tools without adequate psychometric properties in relation to dietary groups might produce significant bias in research.

The ninth study, by Stice et al. [9], reported on the long-term results of an obesity prevention intervention (Project Health) when it was delivered in single-sex or mixed-sex groups combined with food response inhibition and attention training versus single- or mixed-sex groups combined with sham training (i.e., no food response inhibition and attention training). Planet Health, combined with food response inhibition and attention training, significantly reduced body fat over a 2-year follow-up compared to the sham condition regardless of whether the intervention was delivered within single- or mixed-sex groups, although the reduction in fat was more rapid and persistent in the single-sex group. Given these outcomes and considering the positive preventive effect of Planet Health in previous studies, the authors argue that this combined intervention might be a viable option for broad implementation in the future.

The last study in this Special Issue, by Dahill et al. [10], is a prospective study of a community sample of adolescents (*n* = 2056) investigating the association between parents’ positive or negative body shape or weight comments and later symptoms of EDs among their adolescents one year later. They found a positive association between baseline maternal positive comments on eating and adolescents’ eating disorder cognition and quality of life one year later. Interestingly, paternal positive comments on eating were associated with decreased quality of life among the adolescents, while positive paternal comments about weight and shape were associated with a reduction in psychological distress among the adolescents. The thoughtful analyses, including controlling for the stage of adolescents and viewing both positive and negative comments from both mothers and fathers, have been instrumental in providing important nuances in understanding prospective risk factors for the later development of EDs in this paper.

Taken together, the papers included in this Special Issue present a broad range of methods and focus on eating disorders in relation to nutritional beliefs, trends, and practices as well as sex/gender differences. The range in methods is impressive (i.e., from psychometrics, surveys, and cross-sectional correlational studies to longitudinal and experimental studies with both animal models and human subjects). They provide additional pieces of the puzzle of EDs to help us understand the emergence, diagnosis, maintenance, treatment, and prevention of these conditions.
